# Health Tract, No. 142

**Published:** 1863-05

**Authors:** 


### EATING HABITS.

THE most common way to a premature grave, and one of the shortest cuts to that destination is down a man's throat. There is a multitude which no man can number, daily eating immoderately, thus sapping the constitution and laying the foundation for innumerable ills and a too early grave. The wise man does it, and the fool; the virtuous and the abandoned; the kind and the cross, of all climes, are among the errorists. But there are some who are wise as to this point, and the number is increasing; the number of those who are men and women of force; who think for themselves, observe for themselves; who have vigor of intellect enough to compare causes and effects, antecedents and consequents. There is constantly coming to us the knowledge of mothers, who, by the teachings of this JOURNAL, have been led to regulate their households rationally, and are reaping a rich reward in the shape of health for themselves, and what is dearer still, increasing health for their children.

The first great point in the philosophy of eating is to perform that very necessary business with the greatest regularity. A young Scotch trapper, Thomas Glendy, told us thirty years ago, that the Indians, with whom he had been hunting, ate but once a day, and that was in the early evening; that then, a single individual would consume several pounds of meat, smoke his pipe, lie down to sleep, get up by the dawn, hunt all day, eating nothing until the night again. An old beau of Washington City took it into his head that eating was a trouble, and that he would perform that process but once a day. On occasions of his being invited out in the evening, he felt compelled to take something, although he had eaten his regular dinner; but then he would eat nothing at all next day. These irregularities were very rare; he died nearly eighty years of age, a sprightly and gallant old beau to the last. On the other hand, persons who are regularly irregular, seem to live a good while. Captain Hall lately stated to the Historical Society in this city, the case of some Esquimaux, who being carried to sea on a cake of ice, ate absolutely nothing for the space of thirty days, when each man swallowed about thirty pounds of meat and oil, and neither bursted up nor died. But observation has shown that, both as to man and beast, regularity in the hours of eating is indispensable to a healthful, thriving condition. Most articles of food require several hours, to be placed in a condition to be passed out of the stomach; and if a new supply of food is introduced before this process of digestion, of conversion, is completed, the former food is not passed out until the latter has been brought to its own condition; the result of its being kept warm so long is, that it begins to decay, gas is generated, and the whole mass is corrupted. Those who eat often, who eat between meals, always have wind on the stomach, and other places; but if it can not escape, it causes a feeling of weight or oppression, and this is dyspepsia, that horrid hag which has a thousand ails in her train. Half the "girls" have dyspepsia before they are seventeen, in consequence of their everlasting nibbling at every thing in the house. The most natural and healthful times for eating would seem to be at daylight, noon, and sundown; the last meal being very light indeed.

